# Characterization of a New Cyclohexylamine Oxidase From *Acinetobacter* sp. YT-02

**DOI:** 10.3389/fmicb.2018.02848

**Published:** 2018-11-22

**Authors:** Hui Zhou, Zheng-gang Han, Ti Fang, Yuan-yuan Chen, Shang-bo Ning, Ya-ting Gan, Da-zhong Yan

**Affiliations:** ^1^School of Biology and Pharmaceutical Engineering, Wuhan Polytechnic University, Wuhan, China; ^2^State Key Laboratory of Virology, Wuhan Institute of Virology, Chinese Academy of Sciences, Wuhan, China

**Keywords:** cyclohexylamine oxidase, *Acinetobacter* sp. YT-02, cyclohexylamine, cyclohexanone, biodegradation

## Abstract

Cyclohexylamine (CHAM) is widely used in various industries, but it is harmful to human beings and the environment. *Acinetobacter* sp. YT-02 can degrade CHAM via cyclohexanone as an intermediate. In this study, the cyclohexylamine oxidase (CHAO) gene from *Acinetobacter* sp. YT-02 was cloned. Amino acid sequence alignment indicated that the cyclohexylamine oxidase (CHAO_YT–02_) was 48% identical to its homolog from *Brevibacterium oxydans* IH-35A (CHAO_IH–35_). The enzyme was expressed in *Escherichia coli* BL21 (DE3), and purified to apparent homogeneity by Ni-affinity chromatography. The purified enzyme was proposed to be a dimer of molecular mass of approximately 91 kDa. The enzyme exhibited its maximum activity at 50°C and at pH 7.0. The enzyme was thermolabile as demonstrated by loss of important percentage of its maximal activity after 30 min incubation at 50°C. Metal ions Mg^2+^, Co^2+^, and K^+^ had certain inhibitory effect on the enzyme activity. The kinetic parameters *K*_m_ and *V*_max_ were 0.25 ± 0.02 mM and 4.3 ± 0.083 μM min^−1^, respectively. The biochemical properties, substrate specificities, and three-dimensional structures of CHAO_YT–02_ and CHAO_IH–35_ were compared. Our results are helpful to elucidate the mechanism of microbial degradation of CHAM in the strain YT-02. In addition, CHAO_YT–02_, as a potential biocatalyst, is promising in controlling CHAM pollution and deracemization of chiral amines.

## Introduction

Cyclohexylamine (CHAM), an important fine chemical intermediate, is widely used in industrial manufaction of insecticides, antiseptics and plasticizers. In the process of production and usage, CHAM is released into the atmosphere, water, and soil. Furthermore, It was found that sodium N-cyclohexylsulfamate, which is used as a food additive and produced 100,000 tons annually in China, could be decomposed into CHAM by intestinal bacteria (Collings, 1989). CHAM is classified as a volatile organic compound that can enter human body via inhalation or skin contact. CHAM has drawn increasing attention as a weak carcinogen (Price et al., 1970; Petersen et al., 1972). To eliminate CHAM residual dispersed in the environment, certain measures have to be taken. Microbial degradation has advantages of low cost and less energy consumption and is an effective approach to eliminating CHAM pollution and residues in the environment.

To date, limited studies showed that bacteria utilized CHAM as the only source of carbon and nitrogen. These bacteria included *Brevibacterium oxydans* IH-35A (Iwaki et al., 1999a). *Pseudomonas plecoglossicida* NyZ12 (Shen et al., 2008; Yan et al., 2017), and *Acinetobacter* sp. YT-02 isolated by our group (Yan et al., 2018). *Acinetobacter* sp. YT-02 is a Gram-negative bacterium isolated from the activated sludge from a sodium N-cyclohexylsulfamate production plant (Yan et al., 2018). The draft genome showed that *Acinetobacter* sp. YT-02 had poor similarity with cyclohexylamine-degrading bacteria *P. plecoglossicida* NyZ12 (Yan et al., 2017), indicating that our isolate is a new member of CHAM-degrading bacteria (Yan et al., 2018). These strains can convert CHAM into cyclohexanone which was catalyzed by cyclohexylamine oxidase (CHAO) (E.C.1.4.3.12), a type of monoamine oxidases (MAOs). CHAO catalyzes the oxidative deaminnation of cyclohexylamine to form cyclohexanone, hydrogen peroxide and ammonia, using FAD as cofactor (Iwaki et al., 1999b). To date, only CHAO gene from strain *B. oxydans* IH-35A has been cloned, and an X-ray structure of CHAO from *B. oxydans* IH-35A (CHAO_IH–35A_) was determined (Mirza et al., 2013). Although CHAO derived from *Pseudomonas plecoglossicida* NyZ12 was confirmed to be involved in the degradation of cyclohexylamine, there were no relevant biochemical properties reported for this enzyme (Yan et al., 2017). The related biochemical properties for CHAO from *Pseudomonas* sp. (purified enzyme) were investigated, but there is no relevant genetic information (Tokieda et al., 1977). The aim of this study is to clone and heterologously express the CHAO gene from *Acinetobacter* sp. YT-02. Moreover, the enzymatic properties, kinetic parameters, and three-dimensional structure of *Acinetobacter* sp. YT-02 (CHAO_YT–02_) were investigated. The results indicated that CHAO_YT–02_ could be used for elimination of cyclohexylamine in the environment. In addition, CHAO_YT–02_, as a potential biocatalyst in the deracemization of chiral amines, is promising in pharmaceutical industry (Alexeeva et al., 2002; Carr et al., 2003; Leisch et al., 2012; Li et al., 2014; Yao et al., 2018).

## Materials and Methods

### Strains and Plasmids

*Acinetobacter* sp. YT-02 was cultured in minimal salt medium (Yan et al., 2018) using CHAM–HCl (Sinopharm Chemical Reagent, Shanghai, China) at a concentration of 1 g per liter as the sole carbon and nitrogen sources. *Escherichia coli* DH5α and pGEM-T Easy vector were used for DNA cloning. *E. coli* BL21 (DE3) and pET-28b (Novagen, Wisconsin, United States) were used as the host strain and expression vector for gene expression, respectively. When needed, ampicillin and kanamycin were added at final concentrations of 100 and 50 μg/ml, respectively. The restriction enzymes and DNA polymerase were purchased from TaKaRa (Dalian, Liaoning, China). All other chemicals for buffer and medium were of analytical reagent grade.

### Gene Cloning

The genomic DNA of *Acinetobacter* sp. YT-02, extracted using a bacterial DNA kit (OMEGA Bio-Tek, Norcross, United States), was used as the template to amplify the target gene. Primer pair, F1 (5′-TGAATTCGATGAGTGTCAATGACAACCGA-3′) and R1 (5′-CAAGCTTCGAATTGGCCATTGTTTTCT-3′), containing *Eco*RI and *Hin*dIII restriction sites (underlined), respectively, was used for polymerase chain reaction (PCR). PCR amplification was performed as follows: denaturation at 95°C for 5 min, 30 cycles of 95°C for 1 min, 56°C for 1 min, and 72°C for 2 min, and a final extension cycle at 72°C for 8 min. The PCR fragments were cloned into the pGEMT-Easy vector (Promega Corporation, United States) after being added A-tailing using Taq polymerase, and the construct was verified by DNA sequencing. The recombinant plasmid was designated as pGEMT-*chao*. DNA fragments, encoding CHAO (cleaved down from pGEMT-*chao* by *Eco*RI and *Hin*dIII), were inserted into pET-28b, which was digested with the same restriction enzymes, generating the recombinant plasmid pET-28b-*chao*. The correct construct was transformed into *E. coli* BL21 (DE3) competent cell prepared with CCMB80 solution (Hanahan et al., 1991) for gene expression.

### Sequence Analysis

DNA sequence encoding open reading frame (ORFs) of CHAO_YT–02_ was identified by Glimmer 3.02 (Delcher et al., 2007) and ORF finder^1^. Promoter regions and regulatory sites were identified based on neural network promoter prediction^2^. The presence and location of signal peptide were predicted using SignalP server^3^ (Petersen et al., 2011). The amino acid sequence of CHAO_YT–02_ (GenBank accession number: PCN59881) was compared with the protein sequences in GenBank database using BLASTp program with default parameters. Amine oxidase homologs with the highest identity in the database, omitting redundant or ambiguous sequences, were selected for amino acid sequence alignment. The alignment was carried out by ClustalW using MEGA6.0 (Tamura et al., 2013). Phylogenetic tree was created based on the amino acid distances of the aligned sequences using the neighbor-joining method with 1,000 bootstrap replications. Amine oxidase from *Saccharomyces cerevisiae* was used as the outgroup (Landry and Sternglanz, 2003).

### Protein Production and Purification

The recombinant *E. coli* BL21 (DE3) (containing pET-28b-*chao*) cells were cultivated in 4 ml Luria–Bertani (LB) medium (Sambrook et al., 1989) supplemented with kanamycin (50 μg/ml) overnight at 37°C. The culture was inoculated into 200 ml LB medium containing kanamycin (50 μg/ml) and cultured at 37°C. Gene expression was induced by adding isopropy-β-D-thiogalactoside (IPTG) up to a final concentration of 0.2 mM when the OD_600_ of the culture reached 0.8–1.0. After the induction at 18°C for 16 h, the cells were collected by centrifugation. The bacterial pellet was washed twice with phosphate buffer (50 mM, pH 7.0), resuspended in the same buffer. The cells were broken by ultrasonic crusher (parameter settings were as follows: work 6 s, pause 3 s, working time 20 min, and power 400 W), and the supernatant was collected by centrifugation at 12,000 rpm for 30 min at 4°C. The mixture was filtered through a 0.45 μm filter and then loaded onto 1 ml HisTrap column (GE Healthcare, United States) using ÄKTA Prime Plus system. The recombinant protein was eluted with gradient imidazole-containing buffer (50 mM phosphate buffer, 300 mM NaCl, 250 mM imidazole, and pH 7.0). To remove the imidazole and other salt ions in the elute, the protein buffer was changed by using a centrifugal ultrafiltration device (Millipore) with a molecular mass cutoff of 10 kDa. The purity of isolated CHAO_YT–02_ was determined by sodium dodecyl sulfate-polyacrylamide gel electrophoresis (SDS-PAGE) [5% (w/v) stacking gel and 10% (w/v) separating gel] on a vertical mini gel apparatus. The concentration of enzyme was determined by Bradford assay (Bradford, 1976) using bovine serum albumin as a standard.

The molecular mass of CHAO_YT–02_ in solution was determined by gel filtration on Superose^®^ 6 Increase10/300GL column (GE Healthcare, United States). The column was equilibrated with 10 mM phosphate buffer, pH 7.4, containing NaCl 140 mM. The column was calibrated with apoferritin from equine spleen (443 kDa), alcohol dehydrogenase (150 kDa), bovine albumin (66 kDa), and carbonic anhydrase (29 kDa) (Sigma-Aldrich, United States). A linear relationship was established between the *K*_d_ (*K*_d_ = *Ve*/*Vo*, *Ve*: elution volume, *Vo*: void volume) and the logarithm of molecular weight, which was used to calculate the molecular mass of recombinant CHAO_YT–02_.

### Influences of pH and Temperature on the Enzyme Activity and Stability

CHAO_YT–02_ activity was determined using CHAM as the substrate by a modified method (Li et al., 2013). Enzymatic assays were conducted in triplicate. The effect of pH on CHAO_YT–02_ activity was investigated at 30°C in buffers of different pH (sodium acetate for pH 4.0–6.0, sodium phosphate for pH 6.0–8.0, and Tris–HCl for pH 8.0–9.0). The effect of temperature on the activity of CHAO_YT–02_ was measured at pH 7.0. The enzyme was preincubated in buffers of different pH (5.0–9.0) for 30 min at 25°C to determine the pH stability. To determine thermostability, the enzyme was incubated at different temperatures (30–60°C) for 30 min, followed by measurement of the residual activity at 50°C and pH 7.0.

### Determination of Kinetic Parameters

Under the optimal condition (sodium phosphate buffer pH 7.0 and 50°C), CHAO_YT–02_ activity was measured at various CHAM concentrations ranging from 0.1 to 2.5 mM. The *K*_m_ and *V*_max_ were determined by Lineweaver-Burk double-reciprocal plots (Burk et al., 1934).

### Effects of Metal Ions on the Activity of CHAO_YT–02_

To study the effects of metal ions on the activity of CHAO_YT–02_, CuCl_2_, CoCl_2,_ BaCl_2_, KCl, MgCl_2_, NaCl, CaCl_2_, FeCl_2_, MnCl_2_, PbCl_2_, and ZnCl_2_ were used to prepare the metal ion solutions. The enzyme (1.223 mg/ml) was treated with different metal ions at different concentrations (0.5, 1, and 2 mM). The residual activity was assayed at pH 7.0 and 50°C.

### Substrate Spectrum of CHAO_YT–02_

A series of amine substrates (cyclohexylamine, cyclopentylamine, cycloheptylamine, 2-methylcyclohexanamine, 4-methylcyclohexanamine, 1-aminoindane, hexylamine, N-methylcyclohexanamine, 4-methylpiperidine, and N, N-dimethylcyclohexylamine) with diverse structural features were selected to characterize the substrate spectrum of the CHAO_YT–02_. The activity of CHAO_YT–02_ toward each substrate was determined individually at a concentration of 10 mM using the purified enzyme (Tokieda et al., 1977).

### Structure Model of CHAO_YT–02_

The three-dimensional structure model of CHAO_YT–02_ was created by homology modeling using SWISS-MODEL^4^. The crystal structure of CHAO_IH–35A_ was used as the modeling template (Protein Data Bank entry: 4I58). The overall quality of the model was evaluated by MolProbity (Chen et al., 2010). Structural comparison was performed using DaliLite v. 3^5^ (Holm and Laakso, 2016). Structural figures were prepared using PyMOL (Schrödinger).

## Results

### Gene Cloning and Sequence∖Bioinformatics Analysis

Gene *CF596_*10820 in the genome of *Acinetobacter* sp. YT-02 was predicted to encode CHAO_YT–02_ (Yan et al., 2018). A putative promoter region consisting of nucleotides 49–54, TTGAAT (the −35 region) and nucleotides 72–77, TATACT (the −10 region) was present before the coding region (Supplementary Figure S1). The gene contained only one long open reading frame, which encoded a protein of 460 amino acids, as suggested by Glimmer and ORF finder. Signal peptide analysis (Petersen et al., 2011) indicated that no signal peptide was present in the polypeptide chain.

**FIGURE 1:**
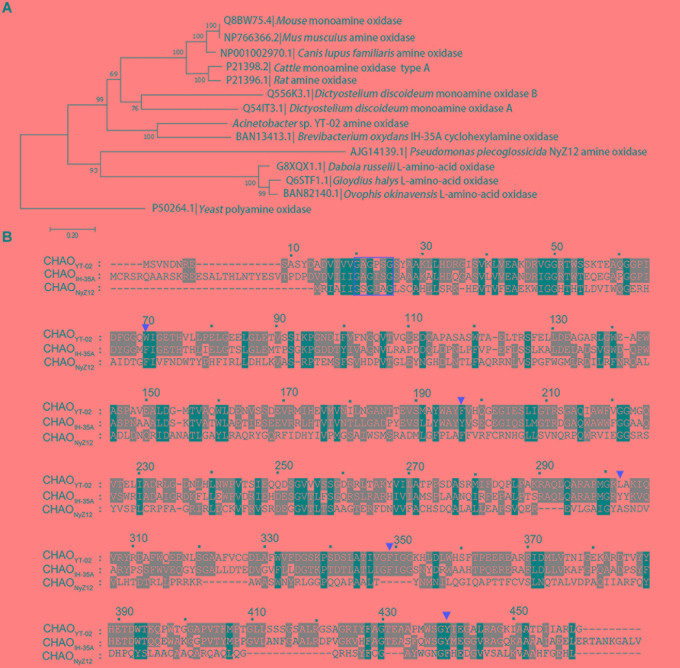
Phylogenetic analysis of cyclohexylamine oxidase (CHAO) and other amine oxidases. **(A)** Phylogenetic tree of CHAO and other amine oxidases. Amino acid sequences were aligned using Clustal W and phylogenetic tree was constructed by using the neighbor joining method using MEGA 6.0. Amine oxidase from *Saccharomyces cerevisiae* was used as the outgroup. The numbers at each branch of phylogenetic tree represent the bootstrap value (1000 replicates). **(B)** Amino acid sequence comparison of CHAO_YT–02_, CHAO_NyZ12_, and CHAO_IH–35A_. A red rectangular box highlights the putative FAD-binding consensus sequence GXGXXG in the N-terminus of CHAOs. Red triangles indicate amino acids in the active center of the enzymes.

The amino-acid sequence of CHAO_YT–02_ was aligned with amine oxidase homologs selected from a protein database. The phylogenetic neighbor-joining tree indicated that CHAO_YT–02_ was closest to CHAO_IH–35A_. These two enzymes shared 48 and 63% amino acid identity and similarity, respectively (Figure 1A). CHAO_YT–02_ showed relatively low amino acid sequence identity and similarity to CHAO_NyZ12_ (17 and 31%, respectively). The amino acids constituting the active-site cavity of CHAO were largely conserved among CHAO_YT–02_, CHAO_IH–35A_, and CHAO_NyZ12_ (Figure 1B). A putative flavin adenine dinucleotide (FAD) binding consensus sequence GXGXXG was present in the N-terminus of CHAOs.

### Expression and Purification of Recombinant CHAO_YT–02_

**FIGURE 2:**
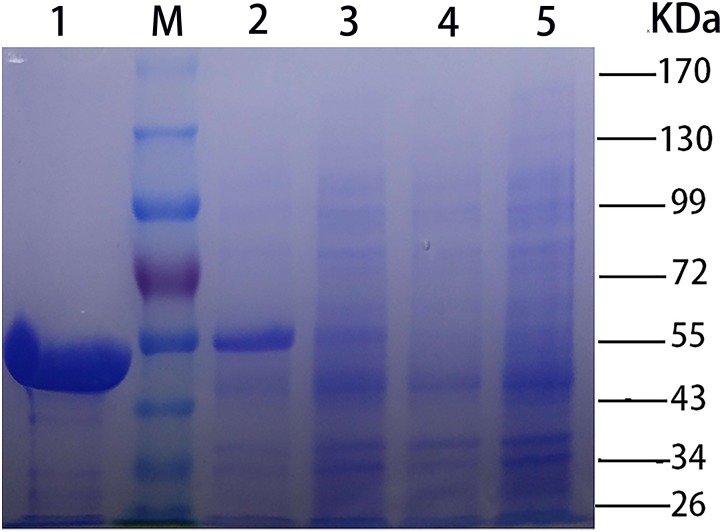
SDS-PAGE [stacking gel (5%, w/v) and separating gel (10%, w/v)] analysis of CHAO_YT–02_. Lanes: M, molecular standard; 1, purified recombinant CHAO_YT–02_; 2, *E. coli* BL21 (DE3) harboring pET-28b-*chao* after induction; 3, *E. coli* BL21 (DE3) harboring pET-28b-*chao* without induction; 4, *E. coli* BL21 (DE3) harboring pET-28b without induction; 5, *E. coli* BL21 (DE3) harboring pET-28b after induction.

The recombinant CHAO_YT–02_ was produced in soluble form in *E. coli* BL21(DE3) at low temperature (18°C). The enzyme was purified by Ni-affinity chromatography. The purified protein showed the expected molecular size of about 55 kDa in SDS-PAGE (Figure 2), consistent with its theoretical molecular mass of 53,534 Da (including 6xHis tag and translated sequence from pET-28b vector). The oligomeric state of purified CHAO was proposed to be a dimer with a molecular mass of approximately 91 kDa as indicated by gel filtration (Supplementary Figure S2).

### Characterization of the Optimum pH and Temperature of CHAO_YT–02_

**FIGURE 3:**
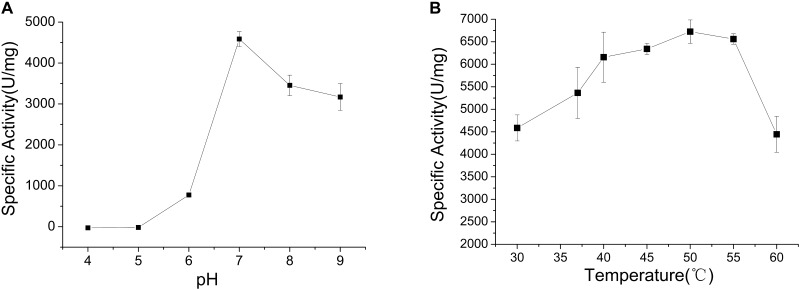
Effects of pH and temperature on the activity of CHAO_YT–02_. **(A)** Effect of pH on CHAO_YT–02_ activity at 30°C. **(B)** Effect of temperature on CHAO_YT–02_ activity.

The optimum pH of CHAO_YT–02_ activity was evaluated by incubating the enzyme in different pH range of 4.0–9.0. The enzyme showed the maximum activity at pH 7.0 and retained more than 60% residual activity at pH 7.0–9.0 (Figure 3A). The effect of temperature on CHAO_YT–02_ activity was measured at different temperatures in the range 30—60°C at pH 7.0. The CHAO_YT–02_ activity increased gradually from 4586 U/mg at 30°C to the maximum of 6724 U/mg at 50°C, and then decreased sharply to 4410 U/mg at 60°C. Therefore, the optimum reaction temperature of CHAO_YT–02_ is 50°C at pH 7.0 (Figure 3B).

### Protein Stability

**FIGURE 4:**
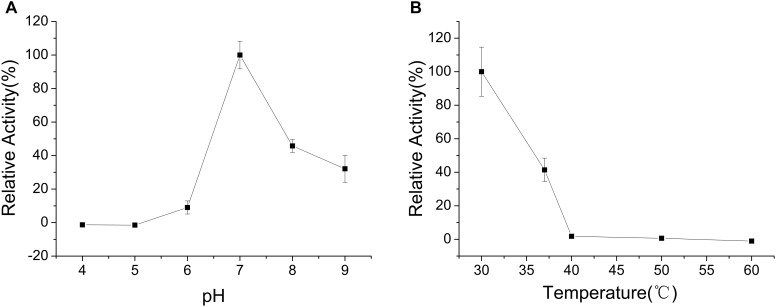
Effects of pH and temperature on the stability of CHAO_YT–02_. **(A)** Effect of pH on the stability of recombinant CHAO_YT–02_. The residual activity was determined at 50°C. The value at 50°C is 100%. **(B)** Effect of temperature on the stability of recombinant CHAO_YT–02_. The residual activity was determined at pH 7.0 (phosphate buffer, 50 mM) and 50°C. The value at pH 7.0 is 100%.

The enzyme was incubated at different pH values (4.0–9.0) for 30 min in the absence of substrate to determine pH stability of CHAO_YT–02_. CHAO_YT–02_ was highly unstable under acidic conditions. The enzyme activity was maintained at more than 50% level after incubation for 30 min at pH 7.0–9.0 (Figure 4A).

To investigate the effect of thermal stability of CHAO_YT–02_, the enzyme was incubated at different temperatures for 30 min in the buffer of pH 7.0. More than 40% activity of the enzyme was retained at 40°C. The activity decreased rapidly when the temperature is higher than 50°C (Figure 4B).

### Effects of Different Metal Ions on the Activity of CHAO_YT–02_

The effects of various metal ions on the activity of CHAO_YT–02_ were determined under standard reaction condition. CHAO_YT–02_ exhibited robust tolerance to several metal ions. Ba^2+^, Na^+^, and Ca^2+^ slightly affected the enzyme activity. Mg^2+^, Co^2+^, and K^+^ led to slight loss of activity (Table 1). The reaction system turned red when the copper ion was added in the absence of enzyme. This unexpected coloration severely disturbed the measurement. The metal ions such as Pb^2+^, Zn^2+^, Fe^2+^, and Mn^2+^ produced precipitation in the reaction system, which hindered the measurement of optical density.

**Table 1:** Effects of metal ions on the activity of CHAO_YT–02_.

Metal ions (2 mM)	Relative activity (%)
None	100 ± 6 (4143 ± 249 U/mg)
Cu^2+^	–
Co^2+^	85 ± 3
Ba^2+^	90 ± 7
K^+^	83 ± 1
Mg^2+^	72 ± 10
Na^+^	102 ± 8
Ca^2+^	104 ± 3
Fe^2+^	–
Mn^2+^	–
Pb^2+^	–
Zn^2+^	–

*–, Not detectable.*

### Kinetic Parameters of CHAO_YT–02_

The dependence of the enzyme reaction rate on substrate was investigated under standard conditions at different concentrations (CHAM, 0.1–2.5 mM). The *K*_m_ and *V*_max_ for the recombinant CHAO_YT–02_ against CHAM at 50°C, pH 7.0 were 0.25 ± 0.02 mM and 4.3 ± 0.083 mMmin^−1^, respectively. The *k*_cat_ and *k*_cat_/*K*_m_ were 523 ± 10 s^−1^ and 2075 s^−1^ mM^−1^ (determined at 50°C), respectively.

### Substrate Spectrum of CHAO_YT–02_

The specific activity of CHAO_YT–02_ toward amines under the standard condition was investigated as shown in Table 2. CHAO_YT–02_ exhibited activity toward a wide range of cycloalkyl primary amine. It showed the highest activity toward CHAM, but also had very low activity toward straight-chain, secondary, and tertiary amines.

**Table 2:** Activity of recombinant CHAO_YT–02_ toward different amine substrates.

Substrate	Structure	Relative activity (%)
Cyclohexylamine	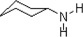	100 ± 6 (4143 ± 249 U/mg)

Cyclopentylamine	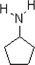	7 ± 2

Cycloheptylamine	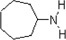	55 ± 2

2-methylcyclohexanamine	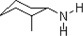	22 ± 2

4-methylcyclohexanamine	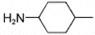	41 ± 3

N-methylcyclohexanamine	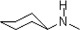	Trace

1-aminoindane	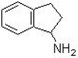	Trace

Hexylamine	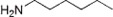	Trace

4-Methylpiperidine	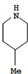	Trace

N, N-dimethylcyclohexylamine	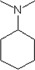	Trace

*The specific activity against different substrates was shown with the relative activity to that of the cyclohexylamine.*

### Structural Model of CHAO_YT–02_

**FIGURE 5:**
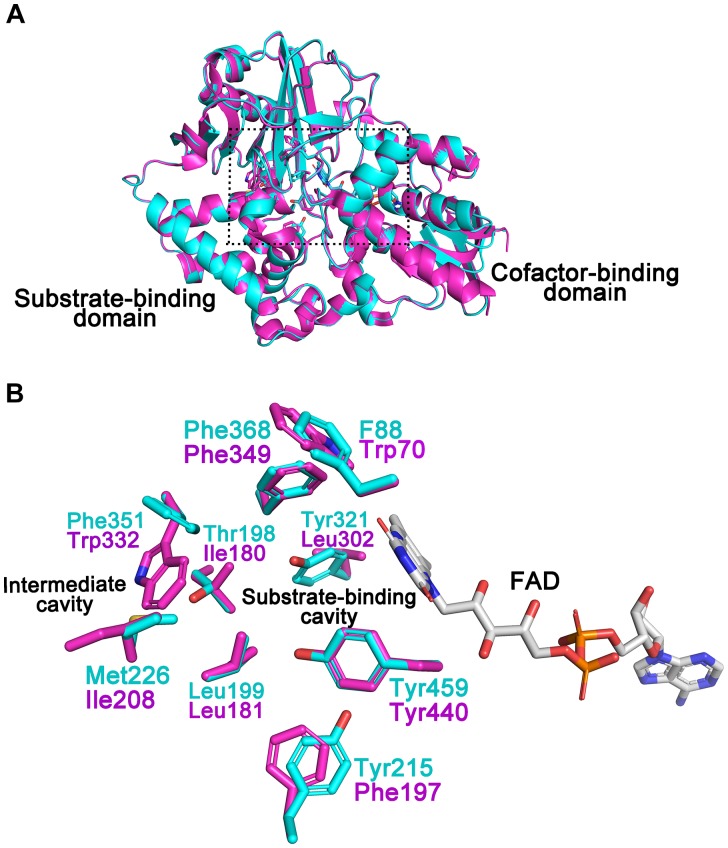
Structural model of CHAO_YT–02_. **(A)** Superposition of the structural model of CHAO_YT–02_ (magenta) and crystal structure of CHAO_IH–35A_ (cyan). A rectangular box indicates the putative positions, where the substrate and the cofactor are located. **(B)** Amino acid compositions of the substrate-binding and intermediate cavities. The carbon atoms in CHAO_YT–02_ and CHAO_IH–35A_ are shown in magenta and cyan, respectively. The carbon atoms in FAD is presented in gray.

A structural model of CHAO_YT–02_ was generated by homology modeling. The overall geometry of the modeled structure displayed a MolProbity of 1.84, thereby indicating good reliability of the theoretical structure. The model of CHAO_YT–02_ consisted of a cofactor-binding domain and a substrate-binding domain (Figure 5A). It showed considerable similarity to the structure of CHAO_IH–35A_, with a root mean square deviation (RMSD) of 0.2 Å. The structure alignment between CHAO_YT–02_ and CHAO_IH–35A_, indicated that a number of amino-acids involved in the substrate-binding (Trp70, Phe197, and Leu302) and substrate/product-traveling (Ile180, Ile208, and Trp332) in CHAO_YT–02_ were different from those in CHAO_IH–35A_ (Figure 5B).

## Discussion

Amine oxidases, a large group of enzymes, are widely present among all kind of organisms (Mondovì and Finazzi Agrò, 1982). In higher organisms, they play an important role in polyamine metabolism, whereas in lower eukaryotes and bacteria amine oxidases are used to provide a source of ammonium (Mirza et al., 2013). CHAO is a member of flavin-containing amine oxidases and is highly specific for catalyzing oxidative deamination of CHAM, forming the oxidation product cyclohexanone (Tokieda et al., 1977; Iwaki et al., 1999a,b; Mirza et al., 2013). The enzyme has great potential in eliminating CHAM pollution (Iwaki et al., 1999b; Yan et al., 2017) and synthesis of chiral amines in pharmaceutical industry (Li et al., 2014; Li et al., 2016). Thus far, only a few CHAOs have been cloned and characterized (Iwaki et al., 1999b; Shen et al., 2008; Mirza et al., 2013; Li et al., 2016; Yan et al., 2017, 2018). The limited enzyme resource cannot satisfy the requirement of their biotechnological applications. Therefore, exploring new CHAO becomes very important. In this study, we reported a new CHAO derived from *Acinetobacter* sp. YT-02. CHAO_YT–02_ was an acid protein with a molecular mass of approximately 55 kDa. Amino acid sequence alignment revealed that CHAO_YT–02_ was approximately 48% identical to the well-characterized CHAO_IH–35A_ obtained from *Brevibacterium oxydans* IH35A.

The optimal temperature for CHAO_YT–02_ activity was 50°C, which was much higher than that of CHAO_IH–35A_ (30°C). However, both the bacterial CHAOs were thermolabile. CHAO_YT–02_ lost almost all activities after a short incubation at 50°C. Both CHAO_YT–02_ and CHAO_IH–35A_ showed their maximum activity at neutral pH (7.0) (Figure 3; Li et al., 2013). However, CHAO_YT–02_ displayed significant portion of the maximum activity under weak basic condition (pH 7-9) and CHAO_IH–35A_ was only active in a very narrow optimal pH range (7.0–7.5) (Iwaki et al., 1999b). Metal ions Mg^2+^, Co^2+^, and K^+^ had certain inhibitory effect on the enzyme activity. With the CHAM as the substrate and with the same reaction temperature (30°C), CHAO_YT–02_ had a considerably smaller *K*_m_ value (0.25 mM) than that of CHAO_IH–35A_ (1.08 mM) (Li et al., 2013). The most notable characteristic of CHAO_YT–02_ was its much higher turnover number (*k*_cat_) (432 s^−1^) than that of CHAO_IH–35A_ (11 s^−1^) (Li et al., 2013). Therefore, CHAO_YT–02_ had a catalytic efficiency 162 times that of CHAO_IH–35A_ (Table 3).

**Table 3:** Kinetic parameters of CHAOs toward CHAM.

Enzymes	*V*_max_ (mM min^−1^)	*K*_m_ (mM)	*k*_cat_ (s^−1^)	*k*_cat_/*K*_m_ (s^−1^ mM^−1^)	References
CHAO_YT–02_	4.30 ± 0.083	0.25 ± 0.02	523 ± 10	2075	This study
(50°C)					
CHAO_YT–02_	2.72 ± 0.065	0.25 ± 0.03	432 ± 10	1724	This study
(30°C)					
CHAO_IH–35A_	ND	1.08 ± 0.14	11.48 ± 0.51	10.63	Li et al., 2013
(30°C)					
CHAO *from*	ND	0.25	ND	ND	Tokieda et al., 1977
*Pseudomonas* sp.					

*ND, Not determined.*

The isolated CHAO_YT–02_ was yellowish, indicating that oxidized form of cofactor FAD incorporated in active-site of the enzymes, consistent with the presence of putative FAD-binding consensus sequence GXGXXG in its N-terminus (Iwaki et al., 1999b; Figure 1B). In the previous study, the crystal structure of CHAO_IH–35A_ in complex with cyclohexanone revealed a number of residues that were responsible for substrate/product interaction (Mirza et al., 2013). Phe88, Tyr215, Tyr321, Phe368, and Tyr459 constituted the substrate-binding cavity located in the interior of protein. The substrate/product was situated in a so-called “aromatic cage,” which consisted of Tyr321, Phe368, Tyr459, and FAD. A similar architecture was present in the modeled structure of CHAO_YT–02_ (Figure 5). Amino acid sequence and structure alignments revealed that these residues were invariant in CHAO_YT–02_, except Tyr321 compared with CHAO_IH–35A_ (Figures 1B, 5). The corresponding residue at the position of Tyr321 in CHAO_YT–02_ was a leucine (Leu302). The leucine substitution causes broader and more hydrophobic cavity for substrate/product in CHAO_YT–02_. In addition, molecular dynamic simulations revealed the presence of an intermediate cavity, which connected the substrate-binding cavity with the exterior of protein. The amino acids, which separated the intermediate and substrate-binding cavities, were largely hydrophobic (Mirza et al., 2013). Evidently, these intermediate cavity gating residues in CHAO_YT–02_ (Ile180, Leu181, Ile208, and Trp332) were more hydrophobic than those in CHAO_IH–35A_ (Thr198, Leu199, Met226, and Phe351) (Figure 5).

Compared with CHAO_IH–35A_, CHAO_YT–02_ displayed a relatively narrow substrate spectrum (Table 2). CHAO_YT–02_ was only active toward primary aliphatic amines with cycloalkane moieties. Similar to CHAO_IH–35A_, CHAO_YT–02_ was most active toward CHAM. Compared with CHAO_IH–35A_, the stronger CHAM-binding affinity (*K*_m_ value) and faster cyclohexanone-releasing (*k*_cat_ value) may be attributed to the more hydrophobicity of the entire substrate/product-binding and -releasing path in CHAO_YT–02_. Site-directed mutagenesis studies showed that mutant T198A displayed enhanced activity relative to wild-type CHAO_IH–35A_ for most (S)-enantiomers of primary amines (Li et al., 2013). The full understanding of enzyme properties and substrate specificity awaits the determination of the crystal structure of CHAO_YT–02_ and complex structures of CHAO_YT–02_ and structurally diverse substrates. A structural study on CHAO_YT–02_ is currently in progress.

## Conclusion

In summary, a novel bacterial CHAO gene from *Acinetobacter* sp. YT-02 was cloned and expressed in *E. coli*. It is the second CHAO that was cloned and comprehensively characterized to date. The phylogenetic analysis indicated that CHAO_YT–02_ is a new member of amine oxidases closely related to CHAO from *B. oxydans* IH-35A. CHAO_YT–02_ showed higher optimal temperature and catalytic efficiency than the well-characterized CHAO_IH–35A_ suggesting the enzyme is more applicable to be used to eliminate the pollutant CHAM in the environmental treatment. In addition, CHAO_YT–02_ was only active toward primary aliphatic amines with cycloalkane moieties which implied a potential application of CHAO_YT–02_ in pharmaceutical industry.

## Author Contributions

DY, HZ, ZH, and TF conceived and designed the experiments. HZ, YC, SN, and YG performed the experiments. HZ, ZH, and TF analyzed the data. HZ, ZH, and DY wrote the paper.

## Conflict of Interest Statement

The authors declare that the research was conducted in the absence of any commercial or financial relationships that could be construed as a potential conflict of interest.

## Footnotes

1 https://www.ncbi.nlm.nih.gov/orffinder/

2 http://www.fruitfly.org/seq_tools/promoter.html

3 http://www.cbs.dtu.dk/services/SignalP/

4 http://swissmodel.expasy.org/

5 http://ekhidna2.biocenter.helsinki.fi/dali/oldstyle.html

## Supplementary Material

FIGURE S1 Nucleotide sequence of the gene *CF596*_10820. A putative promoter region was assigned to nucleotides 49–54 (TTGAAT) for the −35 region, and nucleotides 72–77 (TATACT) for the −10 region. The −10 and −35 regions of the promoter are underlined. The translation start codon for the *CF596*_10820 is shown as the first methionine downstream from T for the transcription start site and GGAGAG for the ribosome binding site (S.D. sequence) (underlined). **(A)** Nucleotide and deduced amino acid sequence of 5′ flanking regions of cyclohexylamine oxidase gene from *Acinetobacter* sp. YT-02. **(B)** Gene sequence chromatogram data for pET-28b-*chao* (sequencing from T7 promoter primer). **(C)** Gene sequence chromatogram data for pET-28b-*chao* (sequencing from T7 terminator primer).

FIGURE S2 Determination of CHAO_YT–02_ molecular mass by gel filtration chromatography. The column was equilibrated with 10 mM phosphate buffer, pH 7.4, containing NaCl 140 mM. The column was calibrated with apoferritin from equine spleen (443 kDa), alcohol dehydrogenase (150 kDa), bovine albumin (66 kDa) and carbonic anhydrase (29 kDa) (Sigma-Aldrich, United States). *V_o_* = 8.049 ml, which was determined by the elution volume of blue dextran (2000 KDa).
