# Supplementary figures and images for: Characterization of a New Cyclohexylamine Oxidase From Acinetobacter sp. YT-02
Source: Front Microbiol. 2018 Nov 22;9:2848. doi: 10.3389/fmicb.2018.02848 (PMC6262902; doi:10.3389/fmicb.2018.02848)

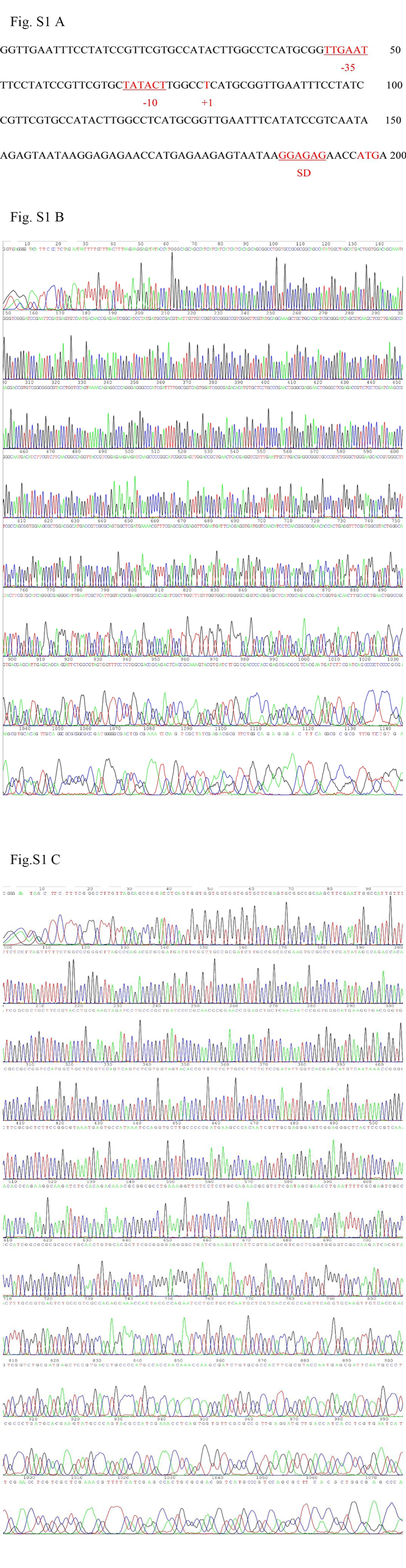

Supplement: FIGURE S1 — Nucleotide sequence of the gene CF596_10820. A putative promoter region was assigned to nucleotides 49–54 (TTGAAT) for the −35 region, and nucleotides 72–77 (TATACT) for the −10 region. The −10 and −35 regions of the promoter are underlined. The translation start codon for the CF596_10820 is shown as the first methionine downstream from T for the transcription start site and GGAGAG for the ribosome binding site (S.D. sequence) (underlined). (A) Nucleotide and deduced amino acid sequence of 5′ flanking regions of cyclohexylamine oxidase gene from Acinetobacter sp. YT-02. (B) Gene sequence chromatogram data for pET-28b-chao (sequencing from T7 promoter primer). (C) Gene sequence chromatogram data for pET-28b-chao (sequencing from T7 terminator primer). [file Image_1.TIF]
